# Clinical Significance of Breast Cancer Molecular Subtypes and Ki67 Expression as a Predictive Value for Pathological Complete Response following Neoadjuvant Chemotherapy: Experience from a Tertiary Care Center in Lebanon

**DOI:** 10.1155/2022/1218128

**Published:** 2022-02-12

**Authors:** Ali Atoui, Maroun Bou Zerdan, Ahmad El Mahmoud, Nathalie Chamseddine, Lina Hamad, Hazem I. Assi

**Affiliations:** ^1^Department of Internal Medicine, Naef K. Basile Cancer Institute, American University of Beirut Medical Center, Beirut, Lebanon; ^2^Faculty of Medicine, American University of Beirut, Beirut, Lebanon

## Abstract

**Introduction:**

Breast cancer is considered nowadays the most prevalent cancer worldwide. The molecular era has successfully divided breast cancer into subtypes based on the various hormonal receptors. These molecular subtypes play a major role in determining the neoadjuvant chemotherapy to be administered. It was noted that the use of neoadjuvant chemotherapy was associated with higher achievement of pathological complete response. The aim of the study was to determine the predictive role of breast cancer subtypes in the efficacy and prognosis of neoadjuvant chemotherapy regimens.

**Methods:**

Combining dose dense anthracycline-based, regular dose anthracycline-based, and nonanthracycline-based chemotherapy, we observed data from 87 patients with breast cancer who received surgery after administration of neoadjuvant chemotherapy at our institution between January 2015 and July 2018. The patients were classified into luminal A, luminal B, HER2 overexpression, and triple negative breast cancer as well as low Ki67 (≤14%) and high Ki67 (>14%) expression groups using immunohistochemistry. Pathologic complete response was the only neoadjuvant chemotherapy outcome parameter. To evaluate variables associated with pathologic complete response, we used univariate analyses followed by multivariate logistic regression.

**Results:**

87 patients with breast cancer were classified into different subtypes according to the 12^th^ St. Gallen International Breast Cancer Conference. The response rate to neoadjuvant chemotherapy was significantly different (*p* = 0.046) between the subgroups. There were significant correlations between pathological complete response (pCR) and ER status (*p* < 0.0001), HER2 (*p* = 0.013), molecular subtypes (*p* = 0.018), T stage (*p* = 0.024), N stage before chemotherapy (*p* = 0.04), and type of chemotherapy (*p* = 0.029). Luminal B type patients had the lowest pCR, followed by luminal A type patients.

**Conclusion:**

Evaluating molecular subtype's significance in breast cancer prognosis warrants additional studies in our region with extensive data about patient-specific neoadjuvant chemotherapy regimens. Our study was able to reproduce results complementary to those present in the literature in other outcomes.

## 1. Introduction

Breast cancer is the most prevalent cancer and is associated with a high mortality rate in females worldwide as well as in the Middle East region. It has been shown in 2013 that breast cancer in Arab women comprises 14-41% of all tumors and is diagnosed on average in 9-50 cases per 100000 women per year while still being on the rise [1].

The use of neoadjuvant chemotherapy (NCT) is associated with higher achievement of pathological complete response (pCR), defined as the absence histologically of cancer cells or their presence in situ in breast tissue with no lymph node involvement [2, 3]. Pathological complete response was found to be associated with a more favorable prognosis in terms of disease-free survival or relapse-free survival in certain molecular subclasses of breast cancer [4–6].

The molecular subtype of the breast cancer constitutes an important prognostic tool when it comes to choosing the most appropriate neoadjuvant chemotherapeutic treatment [4]. In a retrospective study on 240 Chinese women diagnosed with breast cancer, it has been shown that ER and Ki67 expression percentage correlated with pCR rates while age, size of tumor, menstrual status, HER2 overexpression, and metastasis to lymph nodes did not [3]. Additionally, luminal A and B malignancies benefited the least from NCT. Analysis pointed out that higher pCR rates were correlated with the Ki67 expression of more than 40% while the Ki67 expression of less than 40% before chemotherapy was associated with favorable long-term prognosis [3]. Although a recent study has shown excellent survival outcome for patients with hormone-positive, lymph node negative disease, early-stage progesterone receptor negative breast cancer is associated with more aggressive features, poorer outcome, and decreased rate of pCR [7, 8].

In comparison to Europe and the USA, Arab women suffer on average from a more aggressive breast cancer. Indeed, the mean age of presentation of the disease is 10 years earlier for Arab women with the most predominant type being invasive ductal carcinoma [1]. Although breast cancer prevalence is lower than in western countries, higher rates of both inflammatory breast cancers and metastasis to lymph nodes have been noted in the Arab region [1]. Notably, in a chart review of 624 patients presenting with breast cancer between 1990 and 2013 in a Lebanese hospital, 35.4% were luminal A breast cancers while 8.4% were luminal B and 11.6% were triple negative [9]. Moreover, the predominance of the ER positivity in this study conflicts with the Arab standards where the breast cancer tends to be ER receptor negative [9].

In a meta-analysis that included 16 studies with 3776 patients with early-stage breast cancer, administration of NAC before locoregional therapy showed that pathological response is prognostic for relapse-free, disease-free, and overall survival [10].

In this study, we will investigate the clinical significance of breast cancer molecular subtypes including Ki67 on pathological complete response and overall survival, in a tertiary care center in Lebanon. This correlation needs to be further studied in the Middle East region to be able to apply a standardized, yet personalized approach to breast cancer treatment in the region.

## 2. Patients and methods

### 2.1. Patients

In a retrospective study, we included 87 consecutive female breast cancer patients with early-stage disease (stage I, II, III) without evidence of metastasis who were treated at our institution between January 2015 and July 2018. All included patients were aged 18 or more with an Eastern Cooperative Oncology Group (ECOG) score ranging between 0 and 1. Diagnosis of breast cancer was confirmed by pathological examination after core needle biopsies. All patients with a history of previous malignancy were excluded from the study. The research protocol was approved by the medical ethical committee of the American University of Beirut Medical Center.

### 2.2. Chemotherapy

Chemotherapy regimens used were dose dense anthracycline-based, regular dose anthracycline-based, and nonanthracycline-based. Patients with HER2/neu-positive disease received trastuzumab and pertuzumab in the neoadjuvant setting and continued therapy for 1 year after surgery.

### 2.3. Neoadjuvant Chemotherapy (NCT) Outcomes

Primary analytical endpoints were pathological complete response (pCR), partial response (PR), or stable disease .

As mentioned previously, pCR was defined as either no pathological evidence of malignancy or only in situ residuals after surgery with complete disappearance of lymph node metastasis.

### 2.4. Statistical Analysis

Data were entered and analyzed using SPSS version 24.0 (IBM, USA). A two-sided statistical significance was set at a *p* value of 0.05. The univariate associations were computed using Fisher's exact test, bivariate Pearson correlation, and Kruskal Wallis or Mann–Whitney *U* tests as appropriate.

### 2.5. Breast Cancer Classification

Breast cancer staging was done in accordance with the TNM staging system of the American Joint Committee on Cancer (AJCC). Based on the 12th St. Gallen International Breast Cancer Conference (2011) Expert Panel, tumors were classified into five subtypes: luminal A, luminal B, luminal B HER2 type, HER2 overexpression type, and triple negative type [11, 12] (Table 1). Estrogen receptor (ER) and progesterone receptor (PgR) expression through immunohistochemical staining of invasive tumor cells were deemed positive when >10% of tumor cells nuclei were immunoreactive [13]. The HER2 protein expression was defined positive when staining was 3+ in >10% of tumor cells or when the average of HER2 copy number was ≥6.0 signals/cell using fluorescent in situ hybridization (FISH) [14]. Tumor cells were considered FISH negative when the average HER2 copy number was <4.0 signals/cell [14]. As for the Ki67 expression, the average method was used to identify Ki67 positive tumors, following which, the percentages of Ki67-positive tumor cells were calculated. A Ki67 index of ≤14% was defined as low expression, whereas Ki67 index >14% was considered as high expression.

## 3. Results

Among the 87 patients with breast cancer, there were 12 patients (13.8%) with luminal A, 22 patients (25.3%) with luminal B, 23 patients (26.4%) with luminal B HER2 type, 9 patients (10.3%) with triple negative type, and 16 patients (18.3%) with HER2 type. Molecular subtypes could not be determined in 5 patients (5.7%).

There was no significant correlation between age, menstrual status, T and N stage before chemotherapy, and molecular subtypes as shown in Table 2. However, Ki67 expressions with a cut-off of 14% (*p* < 0.0001) were significantly differently distributed between molecular subtypes, being the highest in luminal B subtype. The response rate to neoadjuvant chemotherapy also significantly differed (*p* = 0.046) between the subgroups.

A univariate analysis of clinicopathological indicators and pCR showed no significant correlation between pCR and age, menstrual status, size of tumor, N stage before chemotherapy, ER/PR/HER2 status, expression of Ki67, or type of chemotherapy received (anthracycline-based chemotherapy vs. nonanthracycline-based chemotherapy).

The multivariate logistic regression analysis of the correlation between pCR and clinicopathological features showed significant correlations between pCR and ER status (*p* < 0.0001), HER2 (*p* = 0.013), molecular subtypes (*p* = 0.018), T stage (*p* = 0.024), N stage before chemotherapy (*p* = 0.04), and type of chemotherapy (*p* = 0.029).

Luminal B type patients had the lowest pCR, followed by luminal A type patients (23% in luminal B versus 25% in luminal A). The highest pCR rate was seen in patients with luminal B HER2 type followed by triple negative breast cancer (TNBC) and HER2 type, respectively.

## 4. Discussion

In our study, we analyzed the efficacy of neoadjuvant chemotherapy regimens in 87 breast cancer patients from several countries of the Middle East treated at the American University of Beirut Medical Center in Beirut, Lebanon.

**Figure 1:**
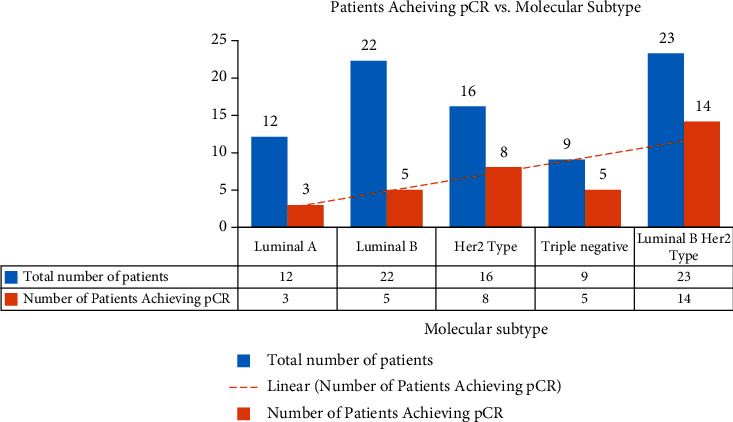
Molecular breast cancer subtypes on the pathological complete response (pCR) rate.

The molecular subtypes associated with the lowest pCR rates were luminal B and luminal A, comprising 23% (5/22) and 25% (3/12), respectively (Figure 1). In a cohort study by Carey et al., pCR for the combined luminal subtypes (luminal A and luminal B) was similarly the lowest among the molecular subtypes identified (7%; *p* = 0.01), and the pCR for triple negative subtype and HER2 subtype was significantly higher (respectively, 27% and 36%, *p* = 0.01) [15]. Carey et al. have found that luminal B subtype was associated with a better pCR compared to luminal A, with 15% of luminal B achieving pCR versus none of luminal A [15]. Our study confirms these findings, where 25% of patients with luminal B versus 23% of patients with luminal A had achieved pCR.

The response to neoadjuvant chemotherapy in our study was significantly higher (*p* = 0.046) in patients with ER/PR negative tumors, such as triple negative type, HER2 overexpression type, and luminal B HER2 molecular subtype. Moreover, patients with luminal A molecular subtype had only 25% (3/12) pCR, and patients with luminal B molecular subtype had 23.8% (5/21) pCR. As mentioned previously, patients with triple negative, HER2 overexpression, and luminal B HER2 molecular subtypes had a remarkable statistically significant response to NCT, with 55.55% (5/9), 50% (8/16), and 60.8% (14/23) achieving pCR, respectively. The previous findings do not fall in line with what is reported in the literature. Hence, in a study conducted by Wang et al., patients with triple negative tumors had a pCR of 23.8%, and patients with HER2 overexpression tumors had a pCR of 22.6%, comprising the highest proportion of patients [3]. However, it is worth noting that Carey et al. revealed that patients achieving pCR had no significant survival benefit compared to those with no pCR regardless of endocrine responsiveness [13]. In this regard, Berruti et al. conducted a meta-regression analysis of 29 heterogenous neoadjuvant trials further proving that pCR is not a surrogate end point for outcomes in patients with breast cancer [16].

When comparing other prognostic factors and as shown in Table 3, age (*p* = 0.615), menstrual status (*p* = 0.664), T stage (*p* = 0.663), and N stage (*p* = 0.374) were not found to be correlated with the percentage of pCR achieved. There was also no statistically significant difference in hormonal status (*p* = 0.05) and Ki67 expression (*p* = 0.606). Lastly, chemotherapy administration was not associated with any significant difference (*p* = 0.911) in achieving pCR. On the other hand, pCR to chemotherapy (*p* = 0.046) and Ki67 expressions with a cut-off threshold of 14% (*p* = 0.0001) were significantly differently distributed between the molecular subtypes being the highest in the luminal B and luminal A, respectively. Breast cancer molecular subtypes in our study sample were equally distributed among age (*p* = 0.339), menstrual status (*p* = 0.6060), T stage (*p* = 0.125), and N stage (*p* = 0.5960). These findings are shown in Table 2. However, after undergoing a multivariate analysis, hormonal status, except for PR (*p* = 0.054), T stage (*p* = 0.024) and N stage (*p* = 0.004) were statistically significant for pCR. Furthermore, upon performing regression analysis, tumor molecular subtype (*p* = 0.018) and dose of anthracyclines (*p* = 0.029) were also statistically significant for pCR.

The Ki67 index is used in routine clinical practice when evaluating breast cancer tumors. Indicating the proliferative rate, it is expressed in all stages of the cycle except the resting phase (G0) [17]. Through immunohistochemistry, cells can be marked, counted, and computed as a percentage of total cells [17]. Tumors with endocrine responsive tumors, HER2 negative expression, and a Ki67 expression ≤ 14% are classified as luminal A subtype [17]. Tumors fitting the first two criteria with a Ki67 expression > 14% are classified as luminal B subtype [17]. In that sense, patients with a high expression of Ki67 have more cells in course of proliferation thus appear to be more sensitive to chemotherapy; hence, patients with high Ki67 expression will likely benefit from chemotherapy. [2, 18]. In addition, these patients are also expected to have higher pCR rates [2, 18]. In a study done by Ohno et al., NCT consisting of fluorouracil, epirubicin, and cyclophosphamide was given to around 477 patients with advanced breast cancer [19]. These patients were then randomized to receive either docetaxel alone or docetaxel and capecitabine. Even though there were no significant differences in the pCR rate (docetaxel/capecitabine: 23%; docetaxel: 24%; *p* = 0.748), patients with a Ki67% greater than 10% had a higher pCR rate than those with a Ki67% less than 10% (12.3% vs. 6.5%, *p* = 0.0004) [19]. Similarly, in our study, 12 patients with luminal A subtypes had a Ki67 expression less than 14%, and none had an expression less than 14% (*p* < 0.0001). This is in line with our previously stated performance description of luminal A subtype. Among these 12 patients, only 3 were able to achieve pCR. In comparison, out of the 22 patients who were identified as having a luminal B molecular subtype, 5 patients achieved pCR. In conclusion, the Ki67 expression might be a factor determining NCT pCR outcome in our study. Additional studies with extensive data about patient specific NCT regimens in our region are warranted to evaluate the prognostic significance of molecular subtype in breast cancer. Even though our findings do not represent novel data, they do provide a real-world experience that serves as an example of a practical clinical application of Ki67 expression profiles in breast cancer in the new and rapidly growing world of molecular diagnostics.

Many factors play a role in explaining the findings in our study. First, our study was conducted retrospectively. Second, the smaller sample size compared to the wider sample size in other studies is a great contributing aspect to the complementary results in our study. Moreover, even though the predictability and prognostication of Ki67 as a biomarker is widely acknowledged, we were unable to investigate them in our study as disease free survival rates were unobtainable due to most patients being lost to follow up [20]. Finally, the relatively incomplete medical history concerning some patients was another limitation of our study. For example, 4 out of 12 patients with no known molecular subtype achieved pCR. Given our small sample size, the distribution of these 4 patients into any one of the four groups could easily yield and obtain other results. Nevertheless, our study was able to reproduce results similar to those present in the literature in other outcomes.

## Figures and Tables

**Table 1:** Molecular breast cancer subtypes based on St. Gallen International Expert Consensus on the Primary Therapy of Early Breast Cancer 2011.

Intrinsic subtype	Clinicopathologic interpretation		
	ER and/or PgR	HER2	Ki67 expression
Lumina A type	Positive	Negative	≤14%
Luminal B type	Positive	Negative	≥ 14%
Luminal B HER2 type	Positive	Positive	≤14% or ≥ 14%
HER2 overexpression type	Negative	Positive	≤14% or ≥ 14%
Triple negative type	Negative	Negative	≤14% or ≥ 14%

**Table 2:** Multivariate logistic regression analysis of the correlation between pCR and clinicopathological features.

	HER2 type	Luminal B	Luminal B HER2 type	Triple negative	Luminal A	*p* value
Age						0.339
≤60	11	20	16	6	10	
>60	5	2	7	3	2	
Menstrual status						0.6060
Premenopause	5	9	7	4	5	
Menopause	8	9	14	5	5	
T stage						0.1250
T1-T2-T3	9	18	20	7	11	
T4	7	4	3	2	1	
N stage						0.5960
N0	1	5	4	0	1	
N1-N2-N3-Nx	15	17	19	9	11	
Ki67						< 0.0001
≤14	6	0	9	0	12	
>14	10	22	14	9	0	
Response to NCT						0.046
Non-pCR	7	16	8	4	9	
pCR	8	5	14	5	3	

**Table 3:** Univariate analysis of clinicopathological indicators and pCR.

	Number of cases	Number of pCR	Percentage of pCR	*p* value
Age				0.615
≤60	67	30	45%	
>60	20	7	35%	
Menstrual status				0.664
Menopause	45	20	44%	
Premenopause	30	11	37%	
T stage				0.663
T1-T2-T3	70	31	44%	
T4	17	6	35%	
N stage				0.374
N0	12	3	25%	
N1-N2-N3-Nx	75	34	45%	
ER				0.461
Negative	27	14	52%	
Positive	60	23	38%	
PR				0.169
Negative	40	22	55%	
Positive	47	15	32%	
HER2				0.151
Negative	47	15	32%	
Positive	39	22	56%	
Ki67				0.606
≤14	30	11	37%	
>14	57	26	46%	
Chemotherapy				0.911
AC	51	23	45%	
No AC	6	3	50%	

## Data Availability

The data used to support the findings of this study are available from the corresponding author upon request.
